# Brief Report: Virtual Reality to Raise Awareness About Autism

**DOI:** 10.1007/s10803-023-06216-y

**Published:** 2023-12-11

**Authors:** Ioulia Koniou, Elise Douard, Marc J. Lanovaz

**Affiliations:** 1 École de psychoéducation, Université de Montréal, P.O. Box 6128, Downtown Station, Montreal, QC H3C 3J7 Canada; 2 Centre de recherche de l’Institut universitaire en santé mentale de Montréal, Montréal, Canada

**Keywords:** Autism, Awareness, Stigmatization, Virtual Reality

## Abstract

**Purpose:**

The purpose of the study was to develop and test a virtual reality application designed to put the participants “in the shoes” of an autistic person during a routine task.

**Method:**

The study involved a randomized controlled trial that included 103 participants recruited from a technical college. Each participant responded to three questionnaires to measure attitudes, knowledge, and openness toward autism. Prior to responding to these questionnaires, the participants in the experimental group also completed an 8-min virtual reality simulation designed by the research team in collaboration with autistic individuals.

**Results:**

The participants who completed the virtual reality simulation reported better attitudes, more knowledge, and higher openness toward autism than the participants in the control group.

**Conclusion:**

The results of the study suggest that virtual reality simulations are promising tools to raise awareness about autism.

**Supplementary Information:**

The online version contains supplementary material available at 10.1007/s10803-023-06216-y.

Although autism has been increasingly present in the news media and television, these portrayals often provide stereotypical and misguided images of autistic individuals to the general population (Jones et al., 2023; Mittmann et al., 2023). These myths and misconceptions may explain why many autistic individuals report facing stigmatization on a daily basis (Crane et al., 2018; Shtayermman, 2009). Stigmatization is characterized by negative attitudes, beliefs, and behaviors that devalue and marginalize individuals, leading to social exclusion (Corrigan & Shapiro, 2010). Researchers have shown that the stigmatization of autistic individuals creates barriers in areas such as education, employment, and healthcare (e.g., Capriola-Hall et al., 2021; Chen et al., 2021; Jones et al., 2021). For instance, autistic students face significant challenges in educational settings, resulting in lower rates of school completion, decreased access to inclusive classrooms and limited availability of support services (Capriola-Hall et al., 2021; Dean & Chang, 2021; Hughes-Roberts et al., 2020; Unigwe et al., 2017).

Similarly, stigmatizing attitudes may lead to discriminatory hiring practices, exclusion from job opportunities, and workplace prejudice (Jones et al., 2021; Roux et al., 2015; Scott et al., 2017). As a result, autistic individuals often face higher rates of unemployment and underemployment compared to their peers, despite their abilities and potential contributions (Scott et al., 2017). Moreover, negative attitudes and stereotypes surrounding autistic individuals may impede access to adequate healthcare services and support (Anderson et al., 2018; Chen et al., 2021; Kinnear et al., 2016).

Previous research has indicated that knowledge about autism and contact with autistic individuals may positively influence attitudes toward this population (Chu et al., 2023; Dachez et al., 2015; Gemegah et al., 2021; Kuzminski et al., 2019). One potential solution to allow people to experience being autistic involves the use of virtual reality (VR). Although VR has shown promising results as a support tool for autistic individuals (e.g., Bravou et al., 2022; Carnett et al., 2023; Lorenzo et al., 2023), researchers have not examined its effects as a tool to raise awareness. That said, some studies have shown that VR perspective-taking experience may result in enhanced prosocial behaviors toward others (e.g., homeless individuals; Herrera et al., 2018; Van Loon et al., 2018). Thus, using virtual reality to put the user “in the shoes” of an autistic person may result in improved perceptions toward this population. To this end, we developed a VR application that allowed participants to experience the perspective of an autistic person during a routine task. Our study aimed to assess the effects of this VR application on attitudes, knowledge, and openness toward autism within a college community.

## Method

### Participants

We recruited individuals from a French-speaking technical college situated in a suburban/rural environment in Canada. The procedures took place directly at a kiosk located on campus. The recruitment of participants was conducted through written advertisements (posters) on billboards across campus and through mass electronic emails directly from the administration of the technical college. Potential participants could either drop by our kiosk, or book an appointment using email or Microsoft Bookings®. To participate in the study, individuals had to be aged 18 years or older, speak and understand French, not have a diagnosis of autism or other pervasive developmental disorders (self-reported), and report that they had never received awareness training on the topic. The study was approved by the institutional review boards of the technical college, of our research center, and of our university. All participants provided informed consent prior to their inclusion in the study. The recruitment occurred from February 21 to February 25, 2022. In total, 103 individuals participated in our research study.

### Virtual Reality Device and Application

In collaboration with autistic individuals, our research team developed a virtual reality application designed to raise awareness about the experience of being autistic. Considering that the main issue lies in the difficulty of people to put themselves “in the shoes” of an autistic person, we simulated potential difficulties that autistic people experience in the sensory and social domains. The application was designed for and installed on a Meta Oculus Quest 2 virtual reality device. The Oculus Quest is a wireless virtual reality headset that does not require the use of a computer, which is affordable, accessible, and easy to use (Bell et al., 2020). The device displays three-dimensional images immersing the user in a virtual environment.

Four autistic individuals participated in a focus group during which the first author asked them a series of questions about their experiences, the most important considerations to address in the application, and ways to simulate autistic characteristics. She also asked the individuals to propose tasks that could be difficult for an autistic person, but easy for a non-autistic person, to complete. Following the focus group, the first and second authors wrote up the tasks to be included in the simulation and sent them back to the autistic individuals for their feedback. This process resulted in two tasks designed to simulate autistic characteristics (see below).

In the application, the user played a virtual character that they could not see directly. The character was an autistic individual who wanted to visit their dentist. In the virtual environment, the user encounters two tasks and has 3 min to complete each one (6 min in total). In the first task, the character is standing outside an elevator on the floor where their dentist office is located. The user must find the office number for their dentist. On the right, there is a letterboard with the office number and the name of every tenant on that floor. To simulate hypersensitivity to external stimuli, the environment overloads the user with visual and auditory distractions such as bright colors, flashing lights, blurry letters, and loud elevator noises. The user can interact with 11 office doors. Attempts at opening incorrect doors lead the user to hearing a voice scolding them for opening the wrong door. The user may try opening multiple doors during the 3-min period. Selecting the correct door leads the user to hear a warm and polite message welcoming them to the dentist’s office. If the user is unable to find the dentist’s office within 3 min, the user is informed that the time has elapsed, and the application moves on to the second task by placing the user directly in the reception area of the office.

**Fig. 1:**
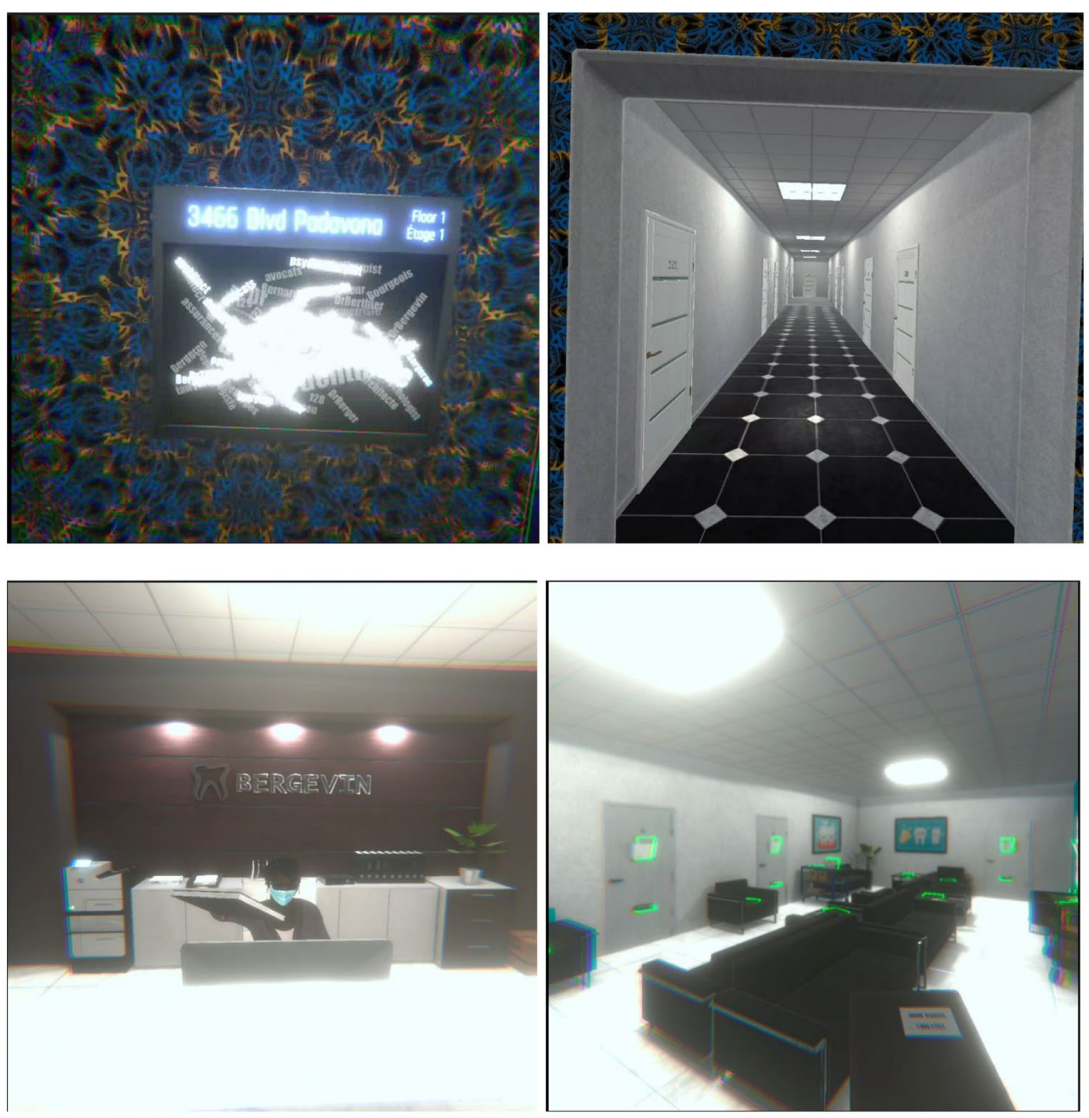
Screenshots of the Application

In the second task, the user is in the reception area and must leave their medical records in a specific location. Once the user picks up their medical file, the receptionist explains where to put it. She uses a specific intonation and provides a facial cue (i.e., wink and smile) to indicate where the file should be left. To simulate challenges associated with nonverbal communication cues, voice intonations were removed and the receptionist wears sunglasses and a mask, which makes the task difficult to carry out. Furthermore, the visual and auditory distractions remain present during this time. The user may place the folder in 21 different locations in the reception area. The user may try multiple locations during the 3-min period. If the user places the folder in the correct location, the receptionist thanks them warmly and politely. Placing the folder in an incorrect location produces an angry comment from the receptionist. If the user is unable to find the correct location for their medical record within 3 min, the user is informed that the time has elapsed. Regardless of the user’s responding, the simulation ends with a 2-min video that summarizes the experience, explains the context, and provides information about autism. Figure 1 presents some screenshots of the application. The virtual reality application is available for download for free at: https://www.oculus.com/experiences/quest/5897385903663059/ (Meta Oculus Quest required) and the English version of the 2-min video at: https://osf.io/e4ad5.

### Measures

To characterize our sample, each participant completed a sociodemographic questionnaire with self-reported items on age, gender, first language learned, citizenship status, student status, education level, domain of study, prior awareness of autism, prior meeting of an autistic individual, and type of connection to autistic individuals.

To assess explicit societal attitudes toward autistic individuals, we used the Societal Attitudes Toward Autism questionnaire (SATA; Flood et al., 2013). Each of the 16 items of the questionnaire has a scale from 0 (Strongly Disagree) to 4 (Strongly Agree) with three items scored in reverse. This instrument has good psychometric values (Flood et al., 2013). The overall score is calculated by summing the items. The lower the score, the more positive the attitudes toward autistic individuals. To assess knowledge about autism, the participants completed the Autism Spectrum Knowledge Scale, General Population version (ASK-GP; McClain et al., 2019). The scale consists of 31 true-or-false items about autism. The overall score is calculated according to the level of difficulty of the item. The higher the score, the more knowledge the person has about autism. The scale has satisfactory psychometric values (McClain et al., 2019). To assess the openness of the participants toward an autistic individual, participants completed the Openness to Autism Scale (OAS; Nevill & White, 2011). The participants read the description of an autistic adult, “Jamie”, although the diagnosis itself is not explicitly mentioned in the description. Then, the questionnaire involved responding to 7 items scaled from 0 (strongly disagree) to 5 (strongly agree) with two items scored in reverse. This instrument has satisfactory psychometric values (Nevill & White, 2011). The overall score is calculated by adding all the items. Higher scores indicate more openness toward autistic individuals. The research team translated each scale to French using a back translation procedure (Weeks et al., 2007). The detailed procedures are described in Koniou et al. (in press).

### Procedures

We conducted a randomized controlled trial where participants were randomly divided into two groups: the experimental group, which used the virtual reality application and responded to the questionnaires, and the control group, which only responded to the questionnaires. Our procedures involved block randomization with each block having a size of four. Two participants in each block were randomly assigned to the control group and the other two to the experimental group. This manipulation prevented the creation of highly imbalanced groups. After obtaining consent and ensuring that the participants met the inclusion criteria, those assigned to the experimental group performed the simulation, which lasted 8 min. To complete the simulation, the participants put on the head-mounted device in an area behind our kiosk, which was enclosed in portable wall panels. Following the simulation, the participants completed the sociodemographic questionnaire (2 min), and our translated French versions of the SATA (5 min), ASK-GP (7 min) and the OAS (3 min). The participants of the control group did not perform the simulation beforehand: they only completed the four questionnaires.

### Statistical Analyses

Our analyses involved the computation of descriptive statistics on the sociodemographic factors and dependent variables for the control and experimental groups. A stepwise algorithm which selected the logistic model with the smallest Akaike information criterion (AIC) examined potential group differences with regards to the sociodemographic information (i.e., age, sex, language, immigration status, employment, education, domain of study, type of connection to autistic individuals and previous knowledge/experience with autism). To estimate the effect size of the VR simulation on societal attitudes, knowledge, and openness toward autism, we used a linear model for each of the scale scores (SATA, OAS, ASK-GP). Each linear regression model included the scale score as main variable, integrated the group as explanatory variable and several sociodemographic measures as confounding factors (age, sex, knowing an autistic individual and previous knowledge/experience with autism). A Bonferroni correction was applied to avoid errors due to multiple testing (significance threshold for the effect-sizes on scale scores = 0.017). All analyses involved the use of R, version 4.1.2. All our code and data are publicly available in an online repository at https://osf.io/qge4y/ (see Supplementary Materials for more details about the analyses).

## Results

**Fig. 2:**
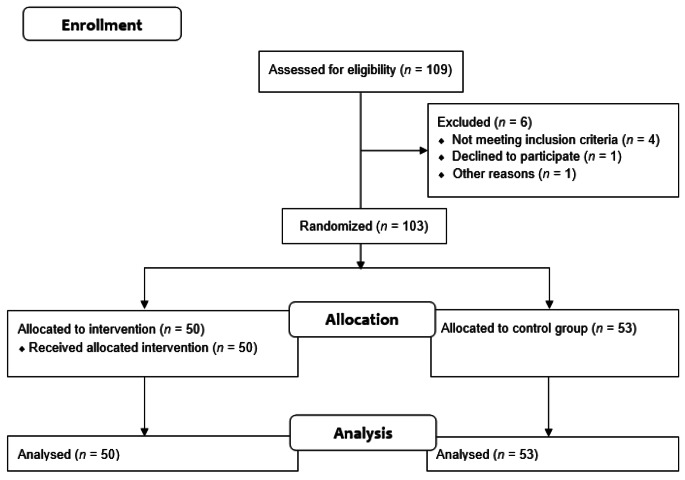
Study CONSORT Diagram

After screening 109 potentially eligible participants, 4 participants were excluded because they were autistic (self-reported), 1 refused to participate and 1 had to be excluded due to a bug in the application (see Fig. 2 for CONSORT diagram). Our sample included a total of 103 participants that had been allocated to either the experimental group (*n* = 50) or the control group (*n* = 53). Table 1 presents the characteristics of the participants. We found no significant group difference concerning the sociodemographic measures included in the logistic regression model selected by the stepwise algorithm with the exception of the domain of study (see Table S1 in the Supplementary Information). The experimental group included sixteen participants studying mathematics, physical, life, and computer sciences whereas the control group only had five. Therefore, we adjusted the following linear models with this covariable.

**Table 1:** Sociodemographic Characteristics of the Participants in Each Group

	Experimental Group (*n* = 50)	Control Group (*n* = 53)
Gender		
Female	27	35
Male	23	15
Other/Prefer not to say	0	3
Age		
18 to 24 years old	41	48
25 to 34 years old	5	4
35 years old and more	4	1
First Language		
French	45	50
English or other	5	3
Status		
Canadian citizen	44	47
Permanent resident or other	6	6
Student Status		
Full-time	46	48
Part-time	2	2
Not a student	2	3
Education		
High school diploma not completed	1	0
High school diploma completed	38	44
College or certificate	8	6
University degree	3	3
Domain		
Arts and humanities	15	24
Health and social sciences	10	7
Mathematics, physical, life, and computer sciences	16	5
Other	9	17
Have you heard of autism?		
Yes	47	52
No	3	1
Have you ever met an autistic individual?		
Yes	41	47
No	9	6
What is your closest connection with this individual?		
None	9	6
Colleague at work or school	14	18
Close friend or child of a close friend	20	16
Family member or live with this individual	7	13

**Fig. 3:**
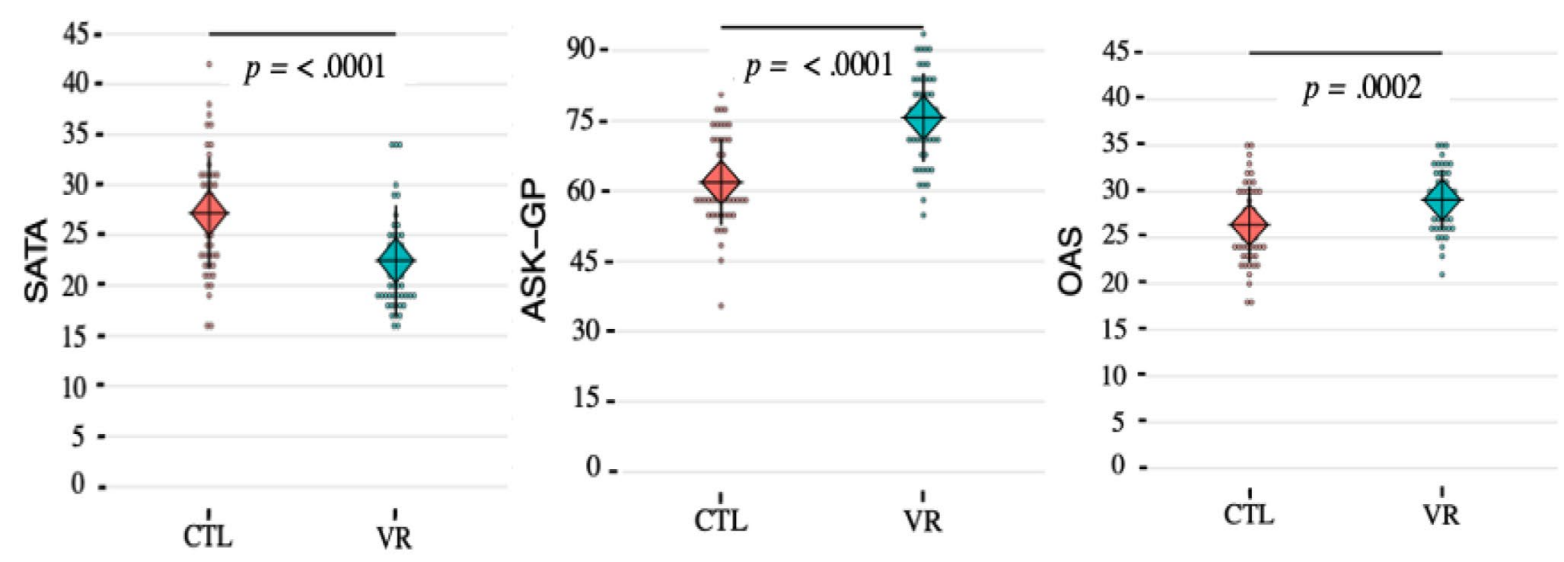
Mean and Distribution of the Outcomes for Each Group *Note*. SATA: societal attitudes toward autism, OAS: openness to autism scale, ASK-GP: autism spectrum knowledge scale, general population version, CTL: controls, VR: experimental group with the VR intervention. Experimental group (*n* = 50); Control group (*n* = 53). Means for each scale score are represented by the cross and the diamonds for the control group (red) and the experimental group (blue). Standard deviations are represented by the vertical black bars. Distributions of scores per individual for each group are represented by the points. The *p*-values correspond to the effect-size of the VR intervention for each score

The linear models showed that the VR simulation had a significant effect on the three measures investigated while adjusting for confounding factors and correcting for multiple statistical tests (see Table 2; Fig. 3). For societal attitudes, the experimental group had a significantly lower average SATA score in comparison to the control group and the VR effect size was of -5.39 points of SATA (*SE* = 1.17, *p* < .0001). When the participant knew an autistic person, particularly a colleague, the score was also significantly lower with an effect size of -4.72 points for the SATA (*SE* = 1.77, *p* = .009), independently of being exposed to the VR simulation or not (see Tables S2 and S3 in the Supplementary Information). Regarding knowledge of autism, the experimental group had a significantly higher average ASK-GP score in comparison to the control group and a VR effect size of 13.58 points (*SE* = 2.05, *p* = < 0.0001). However, knowing an autistic person did not influence the scores on the ASK-GP. Finally, the experimental group had significantly higher OAS scores in comparison to the control group, with a VR effect size of 2.99 points (*SE* = 0.77, *p* = .0002). When the participant knew an autistic person, notably a close friend or a child of a close friend, the score was also significantly higher, independently of being exposed to the VR simulation with an effect size of 4.02 points on the OAS (*SE* = 1.16, *p* = .0008; Tables S2 and S3 in the Supplementary Information).

**Table 2:** Effect Size of the VR Simulation on the Outcomes

Outcomes	Experimental group		Control group		Coefficient	95% CI	*p*
	Mean	*SD*	Mean	*SD*			
SATA	22.54	5.48	27.19	5.53	-5.39	-7.71; -3.06	< 0.0001
ASK-GP	75.68	9.49	61.90	9.15	13.58	9.51; 17.66	< 0.0001
OAS	29.12	3.25	26.36	4.11	2.99	1.47; 4.51	0.0002

*Note*. SATA: societal attitudes toward autism, OAS: openness to autism scale, ASK-GP: autism spectrum knowledge scale, general population version, Experimental group (*n* = 50); Control Group (*n* = 53). Effect-size of the VR intervention on scale score outcomes were predicted by linear models. All models were adjusted for age, sex, knowing an autistic individual and previous knowledge/experience with autism. *p*-values presented are not adjusted for multiple testing (*p*-value threshold after Bonferroni correction = 0.017). SD: standard error, 95% CI: 95% of confidence interval

## Discussion

The results of our study indicate that the VR simulation positively influenced the attitudes, knowledge, and openness of the participants toward autism. These results are consistent with those of prior studies that have used VR to raise awareness about other neurodivergences (Formosa et al., 2018; Tassinari et al., 2022; Yuen & Mak, 2021). A strength of our study is that autistic individuals were involved in the design of the tasks and content included in the virtual reality application. This inclusion may have improved the social validity of the autistic simulation that our participants experienced. One mechanism that may explain the observed changes is that being placed in the shoes of the autistic person may allow the user to develop more empathy toward people with different needs (Lara & Rueda, 2021). That is, prior research had shown that VR perspective-taking experiences may promote prosocial behavior toward people that we consider different (Herrera et al., 2018). Moreover, the informational video embedded within the VR application may have improved knowledge by providing more details and nuance about the condition, which in turn influenced attitudes and openness toward autism in a positive way (Kuzminski et al., 2019; Nevill & White, 2011; Park et al., 2010).

One challenge of using VR to raise awareness is that access to head-mounted devices remains limited in the general population. For this reason, the VR application targets large organizations that train staff to work with autistic individuals or who service this population. For example, universities could use the application to train future professionals (e.g., occupational therapists, behavior analysts), health centers and hospitals could adopt the simulation to raise awareness among their personnel who have contact with autistic individuals (e.g., administrative assistants, nurses), and employers who integrate autistic individuals could raise awareness among managers and co-workers. Albeit promising, VR simulations may not be suitable for everyone. Some parts of the population (e.g., older adults) may experience challenges in moving around in the VR environment, especially if they have never used a joystick in the past.

The pandemic presented unforeseen challenges in conducting the research study. Our original study targeted recruitment of individuals from the general population in a public space. Due to the pandemic, free access to several locations was restricted in our home province, which led us to set up our kiosk in a technical college. Targeting another population may have produced different results. Another limitation of our study was that we did not conduct a long-term follow-up. Hence, whether the observed effects persist over time remains unknown. The lack of any training in our control group prevents us from comparing virtual reality to other ways of raising awareness; that is, whether other types of support tools would be as effective as VR remains an open question. A final limitation of our study is that we relied exclusively on self-report measures to examine our three outcome variables. As such, the extent to which these effects translated to observable behavior changes needs to be further investigated.

Future research should adopt a longitudinal design to track whether these positive outcomes persist. Our virtual reality application simulated a single example of how an autistic person may experience a specific event, going to the dentist. Given the heterogeneity of autistic individuals, future simulations should present a broader range of experiences to prevent the development of stereotypical perceptions. Furthermore, researchers should examine more concrete manifestations of stigmatization (e.g., real-life observations) and the ability of VR to reduce them. Opportunities for social contact and behavioral observations can capture a more natural response and realistic desire for social distance or avoidance, as opposed to self-report scales. Overall, we propose that future research in this area continue to include more individuals to create a sample representative of the population observed over a longer period of time.

## Electronic supplementary material

Below is the link to the electronic supplementary material.

Supplementary Material 1
