# Supplementary material for: Brief Report: Virtual Reality to Raise Awareness About Autism
Source: J Autism Dev Disord. 2023 Dec 11;55(9):3378–86. doi: 10.1007/s10803-023-06216-y (PMC12367809; doi:10.1007/s10803-023-06216-y)
Supplement: Supplementary file 1 — Supplementary Material 1 [file 10803_2023_6216_MOESM1_ESM.pdf]

Supplementary Methods

Statistical Analyses

Stepwise procedures

To investigate a potential group difference for the sociodemographic variables or for the scale items, we used the stepwise algorithm based on AIC available in the stepAIC() function from the MASS package for R (Ripley et al., 2019).

We used this method in a procedure including all the sociodemographic variables (i.e., age, sex, first language, citizenship status, student status, education level, domain of study, knowing an autistic individual, type of connection to autism and previous knowledge/experience with autism).

For this procedure, we included all the explanatory variables to test in a logistic regression as follow:

$$ln(odds(Yi=Group_i)) \sim \beta_0 + \beta_1 \text{ Explanatory variable } 1_i + \ldots + \beta_n \text{ Explanatory variable } n_i$$

Where β_0, β_1 and β_n are the vectors of coefficients for fixed effects. The logistic regression models were computed using the glm() function in R.Then, the stepwise algorithm selected the logistic regression model that best explains the group variance based on the Akaike information criterion (i.e., the one with the smallest AIC).

Linear regression models

To estimate the effect size of the VR intervention on measures of societal attitudes toward autism, knowledge about autism and openness to autism, we used a linear regression model for each of the scale score. Each linear regression model included the scale score as main variable, integrated the group as explanatory variable and several socio-demographic measures as confounding factors (age, sex, knowing an autistic individual and previous knowledge/experience with autism) as follow:

$$\text{Scale Score}_i \sim \beta_0 + \beta_1 \text{ Group}_i + \beta_2 \text{ age}_i + \beta_3 \text{ sex}_i + \beta_4 \text{ knowing an autistic individual}_i + \beta_5 \text{ Prior knowledge about autism}_i$$

Where β_0 to β_5 are the vectors of coefficients for fixed effects. The linear regression models were computed using the glm() function in R.

Supplementary Tables

Table S1

Linear regression including demographic data selected by stepwise procedure

Explanatory variable selected	OR	95% CI	*p*
Control group vs. VR intervention group			
Intercept	0.56	0.26 ; 1.13	0.11
Gender - Male	1.60	0.65 ; 3.96	0.30
Gender - Other	0.00	-	0.99
Study Domain - Health, Social Sciences, Law, or other related domains	2.29	0.72 ; 7.72	0.17
Study Domain - Mathematics, Physical, Life, or Computer Sciences	5.34	1.55 ; 22.00	$\mathbf{1\times10^{-2}}$
Study Domain - Other domains (e.g., Commerce, Administration)	0.84	0.29 ; 2.40	0.75

Note: Best logistic regression model explaining the variances between group selected by an AIC based stepwise algorithm (AIC = 138). Explanatory variable in bold are significant (significant threshold < 0.05). AIC: Akaike information criterion; OR: Odds ratio; 95%CI: 95% confidence interval.

Table S2

Results of the sensitivity analysis including the interaction between group and knowing an autistic individual as predictor of the outcomes

Explanatory variable selected	Coefficient	95% CI	*p*
SATA			
Group × Knowing an autistic individual	-3.82	-9.91 ; 2.27	0.22
OAS			
Group × Knowing an autistic individual	-0.58	-4.58 ; 3.43	0.78

Note: Experimental group (N=50); Control Group (N=53). Effect-sizes on scale score outcomes were predicted by linear models. Models included the same covariate than the main models (i.e., group, knowing an autistic individual, age, sex). P-values were not corrected for multiple testing in the sensitivity analyses. SATA: societal attitudes toward autism, OAS: openness to autism scale, 95%CI: 95% of confidence interval.

Table S3

Results of the sensitivity analysis including the relationship with an autistic individual as predictor of the outcomes

Explanatory variable selected	Coefficient	95% CI	*p*
SATA			
Intercept	30.28	27.25 ; 33.32	1×10^{-35}
Group – VR	-4.92	-7.05 ; -2.78	2×10^{-5}
Relationship – Colleague at work or school	-3.85	-7.20 ; -0.50	3×10^{-2}
Relationship – Close friend or child of a close friend	-3.19	-6.47 ; 0.09	0.06
Relationship – Family member or live with this individual	-3.36	-7.04 ; 0.32	0.08
OAS			
Intercept	23.85	21.83 ; 25.87	2×10^{-41}
Group - VR	2.91	1.49 ; 4.33	1×10^{-4}
Relationship - Colleague at work or school	2.44	0.20 ; 4.67	3×10^{-2}
Relationship - Close friend or child of a close friend	3.14	0.96 ; 5.32	6×10^{-3}
Relationship - Family member or live with this individual	2.98	0.53 ; 5.43	2×10^{-2}

Note: Experimental group (N=50); Control Group (N=53). Effect-sizes on scale score outcomes were predicted by linear models. Models included only the group and the type of relationship as covariate, because no other confounding factors were significant in the previous models. P-values were not corrected for multiple testing in the sensitivity analyses. SATA: societal attitudes toward autism, OAS: openness to autism scale, 95%CI: 95% of confidence interval.

Supplementary References

Ripley B, Venables B, Bates D, et al.: MASS: Support Functions and Datasets for Venables and Ripley's MASS [Internet]2019; Available from: http://www.stats.ox.ac.uk/pub/MASS4/
